# Prior therapeutic experiences and treatment expectations are differentially associated with pain-related disability in individuals with chronic pain

**DOI:** 10.1038/s41598-025-98614-8

**Published:** 2025-04-26

**Authors:** Simon Felix Zerth, Lukas Andreas Basedow, Winfried Rief, Ulrike Bingel, Frank Euteneuer, Jenny Riecke, Marcel Wilhelm, Stefan Salzmann

**Affiliations:** 1 Clinical Psychology and Psychotherapy, Department of Psychology, Philipps University of Marburg, Gutenbergstraße 18, 35032 Marburg, Germany; 2 Center for Translational Neuro- and Behavioral Sciences, Department of Neurology, University Medicine Essen, Hufelandstraße 55, 45147 Essen, Germany; 3 Biological Psychology and Neuroscience, Vinzenz Pallotti University, Pallottistraße 3, 56179 Vallendar, Germany; 4 Medical Psychology, Department of Medicine, Health and Medical University Erfurt, Anger 64- 73, 99084 Erfurt, Germany

**Keywords:** Chronic pain, Treatment experience, Treatment expectation, Psychotherapy, Physiotherapy, Pharmacotherapy, Psychology, Health care

## Abstract

Many individuals suffering from chronic pain do not benefit sufficiently from treatment. Prior treatment experiences and treatment expectations play a significant role in perceived symptom severity and treatment-related outcomes in many chronic diseases. Their role in chronic pain, however, remains underexplored. Therefore, the present study investigated the role of treatment experiences and treatment expectations for pain-related disability in individuals suffering from chronic pain. Participants suffering from chronic pain who were receiving treatment (pharmacotherapy, physiotherapy, and/or psychotherapy) completed questionnaires as part of an online survey. Prior improvement, worsening, and side effect experiences and their relation with treatment expectations were assessed with the *generic rating scale for previous treatment experiences*,* treatment expectations*,* and treatment effects* (GEEE), and pain-related disability via the *pain disability questionnaire* (PDI). Multiple linear regressions were performed to determine how prior treatment experiences related to treatment expectations and whether prior experiences and current treatment expectations were associated with pain-related disability. In total, 212 participants (86.3% female) were included. Prior worsening experience as well as stronger worsening and side effect expectations were associated with higher pain-related disability. Screening patients for different expectation domains could be an important strategy to detect and target potentially relevant factors influencing pain-related disability and treatment outcome.

**Supplementary Information:**

The online version contains supplementary material available at 10.1038/s41598-025-98614-8.

## Introduction

Chronic pain represents one of the most significant health challenges, impacting the lives of millions of people worldwide^1,2^. Despite advances in treating chronic pain, tailored treatments may help improve the available treatments’ efficacy further^3^. When understanding chronic pain from a biopsychosocial perspective^4–6^, it becomes evident that a combination of different treatment approaches may be needed to treat chronic pain adequately. Interdisciplinary and multimodal pain treatments, consisting of pharmacotherapy, physiotherapy, and psychotherapy have been shown to be effective in treating chronic pain conditions^7,8^, even though it is disputed which modalities such multimodal approaches should entail^9^. Since existing treatments do not consistently yield satisfactory outcomes for all patients^10^, precision medicine approaches^3,11^ are needed to further improve treatments. This relates to answering the question of *what works for whom*, in order to ameliorate individual treatment courses^12^. To achieve this, modifiable factors influencing symptom severity have to be identified and addressed. This includes psychological factors^13^, such as (treatment) expectations, which represent one central mechanism of the placebo and nocebo effect^14^, and a powerful predictor of treatment response^15,16^.

Treatment expectations may play an important role in the treatment of a variety of (chronic) diseases^17^, such as Crohn’s Disease, Coronary Heart Disease, and chronic pain^15,18–21^. Optimizing patients’ expectations towards treatment^22^ has been shown to be effective in improving treatment outcomes in various medical conditions^23–25^. For example, meta-analytic data on interventions optimizing expectations of patients with (chronic) pain suggest small to medium effects regarding pain relief^26^. Analogously, negative treatment expectations can contribute to a worsening of symptoms and intensified side effects^27^.

Prior therapeutic experiences represent an important aspect contributing to the formulation of expectations^28,29^ which can influence treatment success^30^. In one recent experimental study by Colloca and colleagues^31^, prior therapeutic experiences predicted placebo effects in both healthy participants and those with chronic orofacial pain, whereas expectation ratings did not. This adds to findings of previous experimental studies on treatment history^32–34^, suggesting that prior treatment experiences may affect treatment outcomes, independent of present expectations.

In the context of chronic pain, the relationship between prior treatment experiences, treatment expectations and relevant patient-reported measures remains underexplored. A better understanding of these factors could help clinicians assess when they should be addressed as part of treatment. This could mean, for example, screening for negative previous treatment experiences and offering a conversation with the aim of managing dysfunctional expectations. Considering that both prior treatment experiences and treatment expectations may independently relate to the course of chronic pain treatment and patient-reported outcomes, the present explorative study aims to elucidate the relationship between prior treatment experiences, treatment expectations, and patient-reported outcomes. More specifically, we aim to investigate (i) how prior experiences with pain treatments and treatment expectations relate to one another, and (ii) which role these factors play in reported pain-related disability.

## Methods

### Procedure

Data were collected online (SoSci Survey, program version 3.3.02) from December 2021 to March 2022 via a database consisting of individuals suffering from chronic pain who either participated in previous studies and/or indicated interest in studies on chronic pain and therefore gave consent to be contacted for study purposes. Individuals who were ≥ 18 years old, who had been suffering from chronic pain for at least 6 months, and whose chronic pain was currently being treated, could participate in the survey. The study was approved by the local Ethics Committee of the Philipps University of Marburg (reference number: 2021-76k) and was conducted in accordance with the Declaration of Helsinki. All participants gave informed consent for their participation in the study.

### Materials

#### Treatment expectations and prior treatment experiences

Patients’ expectations towards pharmacotherapy, physiotherapy, and psychotherapy as treatment modalities for chronic pain, along with their prior experiences with these treatments, were assessed using the generic rating scale for previous treatment experiences, treatment expectations, and treatment effects (GEEE^35^). The GEEE captures treatment expectations, prior treatment experiences as well as current treatment effects regarding improvement, worsening, and side effects on an 11-point Likert-scale (e.g. “how much improvement/worsening/side effects do you expect from the treatment?” from 0, “no expectation of improvement/worsening/side effects” to 10, “expectation of greatest possible improvement/worsening/side effects”). While worsening relates to a worsening of chronic pain symptoms, side effects refer to additional complaints that may occur during treatment. The GEEE can be adapted to different treatment modalities. Evidence from structural equation modeling and correlation analyses with data from six studies that employed the GEEE suggests that the instrument is able to distinctly assess different expectation domains (i.e. improvement, worsening, side effects), suggesting that treatment expectations are not a unitary construct, but rather consist of different expectation domains^36^. Studies regarding the reliability and validity of the instrument are still ongoing.

#### Perceived pain-related disability

The German version^37^ of the pain disability index (PDI^38^) was used to assess pain-related disability. Participants rated their perceived disability from 0 (“no disability”) to 10 (“worst disability”) for seven different domains of functioning and the total item sum was used as a dependent variable in our analysis. The PDI shows good internal consistency, with Cronbach’s α ranging from α = 0.83–0.90^37^.

In addition, participants were asked to indicate pain intensity on an 11-point numerical rating scale, as well as pain duration and location (open-ended questions).

### Statistical analyses

First, to perform multiple linear regression analyses of prior improvement, worsening, and side effect experiences on current expectations for each treatment modality, subsamples were created, which contained participants who indicated having had prior experiences with the respective treatment modality. Multiple linear regression analyses were conducted to assess the influence of prior therapeutic experiences as well as treatment expectations for each treatment modality on pain-related disability. In addition, Bayesian linear regression was conducted to provide a probabilistic framework for model evaluation and predictor inclusion. This approach was chosen for its ability to quantify the strength of evidence for each predictor via Bayes factors and to manage uncertainty in predictor variables, which is particularly relevant in the context of this study. Bayesian linear regression analyses were performed with default model priors as a confirmatory analysis, to further quantify the strength of evidence for the inclusion of predictors. We report Bayes factors BF_M_ for the model with the strongest evidence, representing the change from prior model odds to posterior model odds after observing the data^39^. All analyses were conducted with JASP 0.17.1 (JASP Team, 2023) and *p*-values < 0.05 were considered statistically significant. BF_M_ = 3–10 can be considered substantial, BF_M_ = 10–30 strong and BF_M_ > 30 very strong evidence for the inclusion of one or more predictor(s) to a model^40^.

## Results

### Participants

In total, 262 participants took part in our study. Fifty participants terminated the survey prior to completion and were thus excluded from the analysis. Accordingly, data of *n* = 212 subjects were analyzed. Sample characteristics are presented in Table 1. The sample was predominately female (86.3%), had been suffering from chronic pain for a mean duration of 16.5 years and indicated a mean pain intensity of 7 on an 11-point numerical rating scale for the past week. As current treatment(s) for chronic pain, *n* = 183 participants specified receiving pharmacotherapy, *n* = 113 physiotherapy and *n* = 66 psychotherapy, respectively. A total of *n* = 112 participants stated that they were receiving multiple treatments. As for their past experiences with these treatment modalities, *n* = 189 participants indicated having had experiences with pharmacotherapy, *n* = 186 with physiotherapy, and *n* = 159 with psychotherapy.

**Table 1:** *Sample characteristics*.

Variables	Total sample (N = 212)
Age (y) ^a^	46.1 ± 14.2
Gender, n (% female)	183 (86.3)
Highest educational level, n (%)	
Lower secondary education	51 (24.1)
Higher secondary education	60 (28.3)
Apprenticeship	46 (21.7)
Academic degree	40 (18.9)
Other	15 (7.1)
Employment status, n (%)	
Employed	91 (42.9)
Not employed	121 (57.1)
Pain duration (y) ^a^	16.5 ± 12.3
Pain intensity ^a b^	7.0 ± 1.9
Pain-related disability ^a c^	37.7 ± 14.1
Pain location, n (%) ^d^	
Whole body	92 (43.4)
Back	89 (42.0)
Joint	66 (31.1)
Extremities	59 (27.8)
Abdomen	50 (23.6)
Head	47 (22.2)
Face (e.g. jaw)	28 (13.2)
Other	15 (7.1)
Previous treatment experiences, n (%)	
Pharmacotherapy	189 (89.2)
Physiotherapy	186 (87.7)
Psychotherapy	159 (75.0)
Current treatments, n (%) ^e^	
Pharmacotherapy	183 (86.3)
Physiotherapy	113 (53.3)
Psychotherapy	66 (31.1)

y, years;

^a^Values are presented as means (± standard deviation),

^b^Mean pain intensity during the previous 7 days rated on an 11-point numerical rating scale,

^c^As measured with Pain Disability Index (0–70),

^d^Indication of multiple pain locations was possible,

^e^Indication of multiple treatments was possible.

### Association of prior treatment experiences and treatment expectations

For pharmacotherapy, higher prior improvement experience was only associated with stronger improvement expectations (*b* = 0.52, *t* = 7.53, *p* < .001; Table 2). Higher prior worsening experience was a significant predictor for higher improvement (*b* = 0.29, *t* = 4.11, *p* < .001) and worsening expectations (*b* = 0.24, *t* = 4.24, *p* < .001), while higher side effect experience was only a significant predictor for higher side effect expectations (*b* = 0.20, *t* = 2.90, *p* = .004).

For physiotherapy, higher prior improvement experience was associated with higher improvement expectations (*b* = 0.61, *t* = 12.43, *p* < .001) as well as lower worsening expectations (*b* = -0.10, *t* = -2.44, *p* = .016). Prior worsening experience was only a significant predictor for higher worsening expectations (*b* = 0.46, *t* = 6.09, *p* < .001), while side effect experience was a significant predictor for higher side effect expectations (*b* = 0.83, *t* = 11.98, *p* < .001) as well as for higher worsening expectations (*b* = 0.19, *t* = 2.49, *p* = .014).

For psychotherapy, higher prior improvement experience was associated with higher improvement expectations (*b* = 0.74, *t* = 12.07, *p* < .001) as well as lower worsening expectations (*b* = -0.10, *t* = -2.50, *p* = .013). Higher prior worsening experience was only a significant predictor for higher worsening expectations (*b* = 0.42, *t* = 5.62, *p* < .001). Similarly, higher prior side effect experience was only a significant predictor for stronger side effect expectation (*b* = 0.54, *t* = 6.06, *p* < .001). Regression results are presented in Table 2. Bayesian model comparisons indicated that for each regression model, containing the aforementioned significant predictors was best supported by the data. See Supplemental Tables S1-S9 for Bayesian model comparisons including Bayes factors. Adjusted *R*^2^ values differ substantially between different regression models, ranging between 0.06 ≤ *R*^2^ ≤ 0.57. Particularly, the pharmacotherapy models appear to exhibit a lower goodness of fit, suggesting that the degree of explained variance might differ between treatment modalities.

**Table 2:** *Regressions of associations between prior treatment experiences and current improvement*,* worsening*,* and side effect expectations.*

	Pharmacotherapy subsample		Physiotherapy subsample		Psychotherapy subsample	
Variable	Improvement expectation pharmacotherapy		Improvement expectation physiotherapy		Improvement expectation psychotherapy	
Model fit	*N* = 189, (F(3, 185) = 25.26, *p* < .001, adjusted R^2^ = 0.29)		*N* = 186, (F(3, 182) = 53.37, *p* < .001, adjusted R^2^ = 0.46)		*N* = 159, (F(3, 155) = 54.81, *p* < .001, adjusted R^2^ = 0.51)	
	*b (SE)*	*p*	*b (SE)*	*p*	*b (SE)*	*p*
Prior improvement experience	**0.52 (0.07)**	< 0.001	**0.61 (0.05)**	< 0.001	**0.74 (0.06)**	< 0.001
Prior worsening experience	**0.29 (0.07)**	< 0.001	-0.14 (0.09)	0.127	-0.50 (0.12)	0.667
Prior side effect experience	-0.11 (0.07)	0.138	0.04 (0.09)	0.627	0.11 (0.12)	0.352
Variable	Worsening expectation pharmacotherapy		Worsening expectation physiotherapy		Worsening expectation psychotherapy	
Model fit	*N* = 189, (F(3, 185) = 6.22, *p* < .001, adjusted R^2^ = 0.09)		*N* = 186, (F(3, 182) = 41.55, *p* < .001, adjusted R^2^ = 0.40)		*N* = 159, (F(3, 155) = 48.81, *p* < .001, adjusted R^2^ = 0.47)	
Prior improvement experience	0.01 (0.05)	0.929	**-0.10 (0.04)**	0.016	**-0.10 (0.04)**	0.013
Prior worsening experience	**0.24 (0.06)**	< 0.001	**0.46 (0.08)**	< 0.001	**0.42 (0.07)**	< 0.001
Prior side effect experience	-0.08 (0.06)	0.181	**0.19 (0.07)**	0.014	**0.18 (0.07)**	0.016
Variable	Side effect expectation pharmacotherapy		Side effect expectation physiotherapy		Side effect expectation psychotherapy	
Model fit	*N* = 189, (F(3, 185) = 4.24, *p* < .001, adjusted R^2 ^= 0.06)		*N* = 186, (F(3, 182) = 81.47, *p* < .001, adjusted R^2^ = 0.57)		*N* = 159, (F(3, 155) = 40.11, *p* < .001, adjusted R^2^ = 0.44)	
Prior improvement experience	0.07 (0.06)	0.259	-0.07 (0.04)	0.056	-0.03 (0.05)	0.472
Prior worsening experience	0.01 (0.07)	0.984	-0.06 (0.07)	0.399	0.10 (0.09)	0.273
Prior side effect experience	**0.20 (0.07)**	0.004	**0.83 (0.07)**	< 0.001	**0.54 (0.09)**	< 0.001

Bold values indicate sig. *p* < .05. Prior experiences and current expectations as measured with the GEEE.

### Associations of prior experiences and current expectations with pain-related disability

For pharmacotherapy, higher prior worsening experience (*b* = 1.33, *t* = 3.29, *p* < .001), as well as higher worsening (*b* = 1.46, *t* = 2.49, *p* = .014) and side effect expectation (*b* = 1.36, t = 2.69, *p* = .008) was associated with increased pain-related disability. For physiotherapy, only side effect expectation (*b* = 2.01, *t* = 2.73, *p* = .007) emerged as a significant positive predictor for pain-related disability. For psychotherapy, only worsening expectation (*b* = 1.88, *t* = 2.18, *p* = .031) was positively associated with pain-related disability, see Table 3. Bayesian regression analyses supported these findings, see Supplemental Tables S10-S18.

**Table 3:** Regressions of associations between treatment expectations and pain-related disability.

	Pharmacotherapy subsample		Physiotherapy subsample		Psychotherapy subsample	
Variable	Pain-related disability		Pain-related disability		Pain-related disability	
Model fit	*N* = 189, (F(2, 186) = 0.39, *p* = .679)		*N* = 186, (F(2, 183) = 0.28, *p* = .759)		*N* = 159, (F(2, 156) = 2.09, *p* = .127)	
	*b (SE)*	*p*	*b (SE)*	*p*	*b (SE)*	*p*
Prior improvement experience	-0.21 (0.51)	0.679	-0.18 (0.54)	0.735	-1.14 (0.60)	0.058
Improvement expectation	0.41 (0.46)	0.380	-0.15 (0.60)	0.798	1.05 (0.56)	0.063
Variable	Pain-related disability		Pain-related disability		Pain-related disability	
Model fit	*N* = 189, (F(2, 186) = 11.86, *p* < .001, adjusted R^2^ = 0.10)		*N* = 186, (F(2, 183) = 5.60, *p* = .004, adjusted R^2^ = 0.06)		*N* = 159, (F(2, 156) = 3.94, *p* = .021, adjusted R^2^ = 0.04)	
Prior worsening experience	**1.33 (0.41)**	< 0.001	1.00 (0.66)	0.131	-0.09 (0.70)	0.902
Worsening expectation	**1.46 (0.59)**	0.014	0.99 (0.68)	0.146	**1.88 (0.86)**	0.031
Variable	Pain-related disability		Pain-related disability		Pain-related disability	
Model fit	*N* = 189, (F(2, 186) = 5.11, *p* = .007, adjusted R^2^ = 0.04)		*N* = 186, (F(2, 183) = 7.04, *p* < .001, adjusted R^2^ = 0.07)		*N* = 159, (F(2, 156) = 1.72, *p* = .182)	
Prior side effect experience	0.43 (0.42)	0.306	-0.27 (0.79)	0.733	0.74 (0.69)	0.286
Side effect expectation	**1.36 (0.50)**	0.008	**2.01 (0.74)**	0.007	0.33 (0.74)	0.659

Bold values indicate sig. *p* < .05. Prior experiences and current expectations as measured with the GEEE. Pain-related disability as measured with the PDI.

## Discussion

The present study examined associations of prior treatment experiences and current treatment expectations with pain-related disability in individuals suffering from chronic pain. Our results suggest that treatment experiences are associated with treatment expectations, which is in line with placebo literature^28^. Overall, we could observe a trend indicating that prior treatment experiences are associated with treatment expectations of the same domain (e.g. prior side effect experience is associated with side effect expectation). Although no causal inference can be drawn from cross-sectional investigations, the different time frames indicate that past experiences could determine current treatment expectations. Moreover, stronger worsening and/or side effect expectations seem to be associated with higher pain-related disability, irrespective of treatment modality. Neither having past experience with symptom improvement nor expecting improvement was associated with pain-related disability. This suggests that treatment expectations regarding improvement and those regarding worsening might best be viewed as separate constructs rather than opposite ends of a single spectrum. Results from a recent study analyzing data of different placebo studies support this notion^36^.

The idea that treatment expectations are cognitively represented with more differentiation than a continuum from “positive” to “negative” also bears implications for clinical practice. To best assess patients’ treatment expectations, it might be necessary to not only ask what individuals expect from a treatment but specifically ask for different expectation domains. It seems plausible that multiple expectations regarding a treatment exist simultaneously. This becomes relevant when considering that some expectation domains might not be strongly related to patient-reported outcomes, like it was the case with improvement expectations in our study. Furthermore, when using expectation optimization as an intervention^22^ to ameliorate treatment in patients with chronic pain, a focus on reducing worsening and side effect expectations could be beneficial.

From a clinical perspective, the findings underscore the value of systematically assessing treatment expectations in patients with chronic pain. The Generic Rating Scale for Previous Treatment Experiences, Treatment Expectations, and Treatment Effects (GEEE), as a user-friendly and adaptable tool, could easily be integrated into routine clinical practice. This could help clinicians detect individual maladaptive treatment expectations early and address them through tailored communication or expectation-optimization strategies. Future research could explore the implementation and effectiveness of such screenings in diverse clinical settings. By targeting negative or dysfunctional expectations, such as high worsening or side effect expectations, clinicians may enhance treatment outcomes and reduce pain-related disability. This could represent a feasible strategy to personalize chronic pain treatment. Modifying these expectations could be achieved by offering therapeutic conversations with the aim of reducing potentially harmful treatment expectations, for example by providing realistically-positive information on the treatment. Social observational learning might represent another promising avenue for optimizing treatment expectations in individuals with chronic pain^41,42^.

### Limitations

The data from this study are cross-sectional which limits the degree of interpretation and does not allow for conclusions regarding causality. It might, for example, also be possible that individuals with high disease burden expect more worsening from treatments, since they might have experienced only little treatment benefit in the past. Also, other confounding variables might have led to the associations we found in our data. Since the majority of our sample indicated prior psychotherapy exposure, this might be especially true for constructs such as pain-related self-efficacy, pain catastrophizing, fear-avoidance beliefs, and depressive symptoms, as these variables are found to be associated with pain-related disability^43–45^. A memory bias caused by the current pain experience could also influence reports about past treatment experiences. Therefore, this should be considered a preliminary investigation of the relationship between prior treatment experiences and treatment expectations regarding pain-related disability in individuals with chronic pain. Furthermore, we did not ask the participants specifics about the treatments that they indicated having received in the past or at the time of participation. It might therefore be possible that the data underlying pharmacotherapy, physiotherapy, and psychotherapy are heterogeneous even though they might appear to be unambiguous treatment modalities. This may limit the representativeness of our findings. However, other sample characteristics, such as age and the mean duration of pain, seem to be comparable to other studies investigating treatment expectations in chronic pain populations^46,47^.

Future research could address these limitations by employing longitudinal study designs to establish causal relationships between prior treatment experiences, treatment expectations, and pain-related disability. Additionally, incorporating objective data on treatment types and duration, as well as controlling for potential confounders such as socio-economic status, psychological variables, and access to healthcare, could improve the generalizability and robustness of the findings. To mitigate potential recall bias in reporting past treatment experiences, future studies might consider combining retrospective self-report data with prospective data collection during active treatment phases.

### Conclusions

This is the first study assessing prior treatment experiences as well as treatment expectations in a chronic pain sample for pharmacotherapy, physiotherapy, and psychotherapy. Our results indicate a dimension-specific trend of how treatment experiences are associated with treatment expectations. Moreover, worsening and side effect expectations might play a more important role than improvement expectations regarding pain-related disability in individuals with chronic pain. To personalize and optimize treatment strategies, screening for different expectation domains and reducing pronounced worsening and side effect expectations prior to the beginning of a treatment might be beneficial for individuals suffering from chronic pain.

## Electronic supplementary material

Below is the link to the electronic supplementary material.

Supplementary Material 1

## Data Availability

The datasets used and/or analyzed during the current study are available from the corresponding author upon reasonable request.
