# Supplementary material for: Prior therapeutic experiences and treatment expectations are differentially associated with pain-related disability in individuals with chronic pain
Source: Sci Rep. 2025 Apr 26;15:14687. doi: 10.1038/s41598-025-98614-8 (PMC12033340; doi:10.1038/s41598-025-98614-8)
Supplement: Supplementary file 1 — Supplementary Material 1 [file 41598_2025_98614_MOESM1_ESM.pdf]

Supplemental Table S1. Bayesian model comparison for improvement expectation towards pharmacotherapy

Models	P(M)	P(M|data)	BF_M	BF_{10}	R^2
Prior improvement experience + prior worsening experience	0.083	0.413	7.738	2.122	0.282
Prior improvement experience + prior worsening experience + prior side effect experience	0.250	0.584	4.206	1.000	0.291
Prior improvement experience	0.083	0.003	0.032	0.015	0.225
Prior improvement experience + prior side effect experience	0.083	4.765×10^{-4}	0.005	0.002	0.226
Prior worsening experience	0.083	1.620×10^{-10}	1.782×10^{-9}	8.326×10^{-10}	0.067
Prior worsening experience + prior side effect experience	0.083	5.899×10^{-11}	6.489×10^{-10}	3.032×10^{-10}	0.073
Null model	0.250	6.977×10^{-12}	2.093×10^{-11}	1.195×10^{-11}	0.000
Prior side effect experience	0.083	4.830×10^{-13}	5.313×10^{-12}	2.483×10^{-12}	0.003

Supplemental Table S2. Bayesian model comparison for worsening expectation towards pharmacotherapy

Models	P(M)	P(M|data)	BF_M	BF_{10}	R^2
Prior worsening experience	0.083	0.506	11.258	1.000	0.083
Prior worsening experience + prior side effect experience	0.083	0.227	3.239	0.450	0.092
Prior improvement experience + prior worsening experience + prior side effect experience	0.250	0.163	0.586	0.108	0.092
Prior improvement experience + prior worsening experience	0.083	0.098	1.197	0.194	0.083
Null model	0.250	0.005	0.014	0.003	0.000
Prior side effect experience	0.083	3.283×10^{-4}	0.004	6.490×10^{-4}	0.003
Prior improvement experience	0.083	2.496×10^{-4}	0.003	4.935×10^{-4}	0.000
Prior improvement experience + prior side effect experience	0.083	7.890×10^{-5}	8.680×10^{-4}	1.560×10^{-4}	0.004

Supplemental Table S3. Bayesian model comparison for side effect expectation towards pharmacotherapy

Models	P(M)	P(M|data)	BF_M	BF_{10}	R^2
Prior side effect experience	0.083	0.499	10.961	1.000	0.058
Prior improvement experience + prior side effect experience	0.083	0.187	2.530	0.375	0.064
Prior improvement experience + prior worsening experience + prior side effect experience	0.250	0.142	0.498	0.095	0.064
Prior worsening experience + prior side effect experience	0.083	0.103	1.261	0.206	0.058
Null model	0.250	0.050	0.158	0.033	0.000
Prior worsening experience	0.083	0.009	0.103	0.019	0.014
Prior improvement experience	0.083	0.005	0.060	0.011	0.008
Prior improvement experience + prior worsening experience	0.083	0.004	0.045	0.008	0.022

Supplemental Table S4. Bayesian model comparison for improvement expectations towards psychotherapy

Models	P(M)	P(M|data)	BF_M	BF_{10}	R^2
Prior improvement experience	0.083	0.766	35.991	1.000	0.511
Prior improvement experience + prior side effect experience	0.083	0.112	1.381	0.146	0.514
Prior improvement experience + prior worsening experience	0.083	0.079	0.947	0.104	0.512
Prior improvement experience + prior worsening experience + prior side effect experience	0.250	0.043	0.136	0.019	0.515
Prior side effect experience	0.083	1.457×10^{-22}	1.603×10^{-21}	1.903×10^{-22}	0.058
Prior worsening experience + prior side effect experience	0.083	3.307×10^{-23}	3.638×10^{-22}	4.318×10^{-23}	0.059
Null model	0.250	3.126×10^{-23}	9.377×10^{-23}	1.360×10^{-23}	0.000
Prior worsening experience	0.083	1.865×10^{-23}	2.051×10^{-22}	2.435×10^{-23}	0.032

Supplemental Table S5. Bayesian model comparison for worsening expectations towards psychotherapy

Models	P(M)	P(M|data)	BF_M	BF_{10}	R^2
Prior improvement experience + prior worsening experience + prior side effect experience	0.250	0.692	6.748	1.000	0.475
Prior worsening experience	0.083	0.116	1.443	0.503	0.440
Prior improvement experience + prior worsening experience	0.083	0.102	1.256	0.444	0.455
Prior worsening experience + prior side effect experience	0.083	0.089	1.078	0.387	0.454
Prior improvement experience + prior side effect experience	0.083	1.418×10^{-6}	1.560×10^{-5}	6.147×10^{-6}	0.369
Prior side effect experience	0.083	1.076×10^{-6}	1.184×10^{-5}	4.664×10^{-6}	0.349
Null model	0.250	1.816×10^{-19}	5.449×10^{-19}	2.624×10^{-19}	0.000
Prior improvement experience	0.083	1.067×10^{-20}	1.174×10^{-19}	4.625×10^{-20}	0.000

Supplemental Table S6. Bayesian model comparison for side effect expectations towards psychotherapy

Models	P(M)	P(M|data)	BF_M	BF_{10}	R^2
Prior side effect experience	0.083	0.699	25.518	1.000	0.431
Prior worsening experience + prior side effect experience	0.083	0.134	1.709	0.192	0.435
Prior improvement experience + prior side effect experience	0.083	0.096	1.169	0.137	0.433
Prior improvement experience + prior worsening experience + prior side effect experience	0.250	0.071	0.228	0.034	0.437
Prior worsening experience	0.083	1.315×10^{-7}	1.446×10^{-6}	1.882×10^{-7}	0.304
Prior improvement experience + prior worsening experience	0.083	1.825×10^{-8}	2.007×10^{-7}	2.611×10^{-8}	0.304
Null model	0.250	3.707×10^{-18}	1.112×10^{-17}	1.768×10^{-18}	0.000
Prior improvement experience	0.083	7.828×10^{-19}	8.611×10^{-18}	1.120×10^{-18}	0.018

Supplemental Table S7. Bayesian model comparison for improvement expectations towards physiotherapy

Models	P(M)	P(M|data)	BF_M	BF_{10}	R^2
Prior improvement experience	0.083	0.625	18.336	1.000	0.460
Prior improvement experience + prior worsening experience	0.083	0.214	2.987	0.342	0.467
Prior improvement experience + prior worsening experience + prior side effect experience	0.250	0.085	0.280	0.046	0.468
Prior improvement experience + prior side effect experience	0.083	0.076	0.904	0.122	0.461
Null model	0.250	1.621×10^{-23}	4.864×10^{-23}	8.647×10^{-24}	0.000
Prior worsening experience	0.083	3.602×10^{-24}	3.962×10^{-23}	5.763×10^{-24}	0.017
Prior side effect experience	0.083	1.620×10^{-24}	1.782×10^{-23}	2.592×10^{-24}	0.007
Prior worsening experience + prior side effect experience	0.083	8.305×10^{-25}	9.136×10^{-24}	1.329×10^{-24}	0.017

Supplemental Table S8. Bayesian model comparison for worsening expectations towards physiotherapy

Models	P(M)	P(M|data)	BF_M	BF_{10}	R^2
Prior improvement experience + prior worsening experience + prior side effect experience	0.250	0.754	9.193	1.000	0.406
Prior worsening experience + prior side effect experience	0.083	0.108	1.326	0.428	0.387
Prior improvement experience + prior worsening experience	0.083	0.096	1.164	0.381	0.386
Prior worsening experience	0.083	0.043	0.491	0.170	0.365
Prior improvement experience + prior side effect experience	0.083	1.283×10^{-7}	1.411×10^{-6}	5.105×10^{-7}	0.285
Prior side effect experience	0.083	7.143×10^{-8}	7.857×10^{-7}	2.842×10^{-7}	0.264
Null model	0.250	2.312×10^{-18}	6.937×10^{-18}	3.067×10^{-18}	0.000
Prior improvement experience	0.083	2.181×10^{-18}	2.400×10^{-17}	8.680×10^{-18}	0.033

Supplemental Table S9. Bayesian model comparison for side effect expectations towards physiotherapy

Models	P(M)	P(M|data)	BF_M	BF_{10}	R^2
Prior side effect experience	0.083	0.567	14.428	1.000	0.563
Prior improvement experience + prior side effect experience	0.083	0.262	3.898	0.461	0.571
Prior improvement experience + prior worsening experience + prior side effect experience	0.250	0.110	0.370	0.065	0.573
Prior worsening experience + prior side effect experience	0.083	0.061	0.716	0.108	0.565
Prior worsening experience	0.083	1.563×10^{-23}	1.719×10^{-22}	2.755×10^{-23}	0.223
Prior improvement experience + prior worsening experience	0.083	1.040×10^{-23}	1.144×10^{-22}	1.832×10^{-23}	0.237
Null model	0.250	6.318×10^{-32}	1.896×10^{-31}	3.712×10^{-32}	0.000
Prior improvement experience	0.083	2.031×10^{-32}	2.235×10^{-31}	3.580×10^{-32}	0.021

Supplemental Table S10. Bayesian model comparison for prior pharmacotherapeutic improvement experience and improvement expectation on pain-related disability.

Models	P(M)	P(M|data)	BF_M	BF_{10}	R^2
Null model	0.333	0.808	8.430	1.000	0.000
Improvement expectation	0.167	0.085	0.462	0.209	0.003
Prior improvement experience	0.167	0.064	0.340	0.158	0.000
Prior improvement experience + improvement expectation	0.333	0.043	0.091	0.054	0.004

Supplemental Table S11. Bayesian model comparison for prior pharmacotherapeutic worsening experience and worsening expectation on pain-related disability.

Models	P(M)	P(M|data)	BF_M	BF_{10}	R^2
Prior worsening experience + worsening expectation	0.333	0.858	12.041	1.000	0.113
Prior worsening experience	0.167	0.127	0.725	0.295	0.084
Worsening expectation	0.167	0.015	0.076	0.035	0.061
Null model	0.333	7.174×10^{-4}	0.001	8.366×10^{-4}	0.000

Supplemental Table S12. Bayesian model comparison for prior pharmacotherapeutic side effect experience and side effect expectation on pain-related disability.

Models	P(M)	P(M|data)	BF_M	BF_{10}	R^2
Side effect expectation	0.167	0.517	5.358	1.000	0.047
Prior side effect experience + side effect expectation	0.333	0.356	1.107	0.344	0.052
Null model	0.333	0.097	0.215	0.094	0.000
Prior side effect experience	0.167	0.029	0.150	0.056	0.015

Supplemental Table S13. Bayesian model comparison for prior physiotherapeutic improvement experience and improvement expectation on pain-related disability.

Models	P(M)	P(M|data)	BF_M	BF_{10}	R^2
Null model	0.333	0.802	8.111	1.000	0.000
Prior improvement experience	0.167	0.080	0.435	0.200	0.003
Improvement expectation	0.167	0.078	0.424	0.195	0.002
Prior improvement experience + improvement expectation	0.333	0.039	0.082	0.049	0.003

Supplemental Table S14. Bayesian model comparison for prior physiotherapeutic worsening experience and worsening expectation on pain-related disability.

Models	P(M)	P(M|data)	BF_M	BF_{10}	R^2
Prior worsening experience + worsening expectation	0.333	0.349	1.072	1.000	0.058
Prior worsening experience	0.167	0.307	2.211	1.757	0.047
Worsening expectation	0.167	0.283	1.972	1.621	0.046
Null model	0.333	0.062	0.131	0.176	0.000

Supplemental Table S15. Bayesian model comparison for prior physiotherapeutic side effect experience and side effect expectation on pain-related disability.

Models	P(M)	P(M|data)	BF_M	BF_{10}	R^2
Side effect expectation	0.167	0.676	10.448	1.000	0.071
Prior side effect experience + side effect expectation	0.333	0.287	0.805	0.212	0.071
Prior side effect experience	0.167	0.022	0.114	0.033	0.034
Null model	0.333	0.014	0.029	0.011	0.000

Supplemental Table S16. Bayesian model comparison for prior psychotherapeutic improvement experience and improvement expectation on pain-related disability.

Models	P(M)	P(M|data)	BF_M	BF_{10}	R^2
Null model	0.333	0.663	3.940	1.000	0.000
Prior improvement experience + improvement expectation	0.333	0.188	0.463	0.283	0.026
Prior improvement experience	0.167	0.077	0.416	0.231	0.004
Improvement expectation	0.167	0.072	0.388	0.217	0.003

Supplemental Table S17. Bayesian model comparison for prior psychotherapeutic worsening experience and worsening expectation on pain-related disability.

Models	P(M)	P(M|data)	BF_M	BF_{10}	R^2
Worsening expectation	0.167	0.529	5.610	1.000	0.048
Prior worsening experience + worsening expectation	0.333	0.243	0.644	0.230	0.048
Null model	0.333	0.169	0.407	0.160	0.000
Prior worsening experience	0.167	0.059	0.312	0.111	0.019

Supplemental Table S18. Bayesian model comparison for prior psychotherapeutic side effect experience and side effect expectation on pain-related disability.

Models	P(M)	P(M|data)	BF_M	BF_{10}	R^2
Null model	0.333	0.545	2.398	1.000	0.000
Prior side effect experience	0.167	0.209	1.321	0.766	0.020
Side effect expectation	0.167	0.134	0.774	0.492	0.014
Prior side effect experience + Side effect expectation	0.333	0.112	0.252	0.205	0.022
